# Clinical outcomes and toxicity in metastatic breast cancer patients treated with first or second-line cyclin-dependent kinase (CDK) 4/6 inhibitors and endocrine therapy in Egypt

**DOI:** 10.11604/pamj.2026.53.121.49258

**Published:** 2026-03-11

**Authors:** Hesham Mahmoud ElWakeel, Hagar Ibrahim Elghazawy, Nagy Samy Gobran, Asmaa Ali Kortoma

**Affiliations:** 1 Clinical Oncology and Nuclear Medicine Department, Ain Shams University, Cairo, Egypt

**Keywords:** Cyclin-dependent kinase 4/6 inhibitors, hormone receptor-positive/human epidermal growth factor receptor 2-negative, metastatic breast cancer, efficacy, toxicity

## Abstract

**Introduction:**

cyclin-dependent kinase 4/6 inhibitors (CDK4/6is) are widely used to treat hormone receptor-positive/human epidermal growth factor receptor 2-negative (HR+/HER2-) metastatic breast cancer (MBC). This study aimed to assess the real-world efficacy and tolerability of CDK 4/6i in the Egyptian population.

**Methods:**

prospective observational study for HR+/HER2- MBC treated in the clinical oncology department of Ain Shams University Hospitals, Egypt, between July 2022 and September 2024.

**Results:**

sixty-two patients were included (mean age, 53.8 years; range, 29-85). Thirty-one (50%) received CDK4/6is as first-line therapy and 31 as second-line therapy. Median follow-up time was 14.2 months (range 12-24 months). Thirty patients (48.3%) started on Ribociclib, 9 (14.5%) on Abemaciclib, and 23 (37.1%) on Palbociclib, with 15 patients switched from Ribociclib and Abemaciclib to Palbociclib due to logistical causes. Median progression-free survival (PFS) was 14 months (6-27 months) and 11 months for first- and second-line therapy, respectively. Patients and treatment characteristics did not significantly impact PFS. Ribociclib and Palbociclib most frequently caused neutropenia (70.0% and 68.4%, respectively), while Abemaciclib caused diarrhea (55.6%). The rate of dose reductions was 24.6%. Age was significantly correlated with a higher incidence of hematological (p=0.03) and nephrological toxicity (p=0.02). Furthermore, medical comorbidities were associated with a significantly higher frequency of grade 3-4 hematological events (p=0.02).

**Conclusion:**

our findings demonstrate that CDK4/6is are both effective and well-tolerated for HR+//HER2- MBC patients within a diverse patient population. Additionally, this prospective evaluation supports the efficacy of lower-dose levels of CDK4/6is, without negatively impacting PFS.

## Introduction

Breast cancer (BC) is the most common malignancy and the leading cause of cancer-related mortality among women globally [1]. Metastatic breast cancer (MBC) is considered incurable, with treatment objectives focused on controlling disease progression, prolonging survival, and improving quality of life [2]. The estimated 5-year survival rate remained discouraging at approximately 25% despite advancements in therapeutic strategies over the past decade [3]. Treatment decisions in MBC are influenced by various factors, including hormone receptor (HR) and human epidermal growth factor receptor 2 (HER2) status. Approximately 70% of BC cases are HR+/HER2 [4], making endocrine therapy (ET) the primary treatment modality for this subgroup. Nearly 50% of MBC patients develop resistance to ET [5]. To overcome this, targeted therapies such as cyclin-dependent kinase 4/6 inhibitors (CDK4/6is) have been approved for HR+/HER2- MBC [5,6]. Palbociclib, Ribociclib, and Abemaciclib -approved in combination with ET became the standard of care in the first-line setting (Abemaciclib is also approved as monotherapy following MONARCH-1 results [7]) for HR+/HER2- MBC, based on PALOMA-2 (2016), MONALEESA-2 (2017), and MONARCH-3 (2018) respectively, which showed significant improvements in progression-free survival (PFS) and overall survival (OS) compared to ET alone [8]. Despite the clear clinical benefit shown in randomized controlled trials (RCTs), real-world data are needed to assess the combination´s efficacy and safety in routine practice, as some patient subgroups are underrepresented or excluded from major clinical trials, such as the elderly, patients with comorbidities, Eastern Cooperative Oncology Group (ECOG) performance status (PS) >1, or CNS metastases. Real-world data (RWD) can therefore provide valuable insights into treatment outcomes in these populations [9].

In this context, we conducted a prospective observational study of women with HR+/HER2- MBC, who received CDK4/6is in combination with ET, at the Department of Clinical Oncology, Ain Shams University Hospitals, Egypt. Our aim was to evaluate clinical efficacy (PFS) and toxicity in routine practice. A secondary aim was to assess the impact of clinicopathological factors, type of CDK4/6is, and dose modifications on treatment outcomes.

## Methods

**Study design:** a prospective observational cohort study conducted to investigate the efficacy and safety of CDK4/6is in the treatment of HR+/HER2- MBC. Eligible patients were enrolled at the time of treatment decision and followed prospectively until disease progression, death, or the end of the study period.

**Study setting:** the study was conducted at the Department of Clinical Oncology, Ain Shams University Hospitals, Cairo, Egypt. Recruitment period was from July 2022 to July 2023, and prospective follow-up continued until September 2024.

**Participants:** all female patients aged 18 years or older with confirmed HR+/HER2- MBC, with an ECOG performance status (PS) of 0 to 3, adequate hematological and organ functions (patients with medical co-morbidities were allowed) and planned to receive CDK4/6is in combination with ET as either first- or second-line therapy following prior ET line.

**Figure 1:**
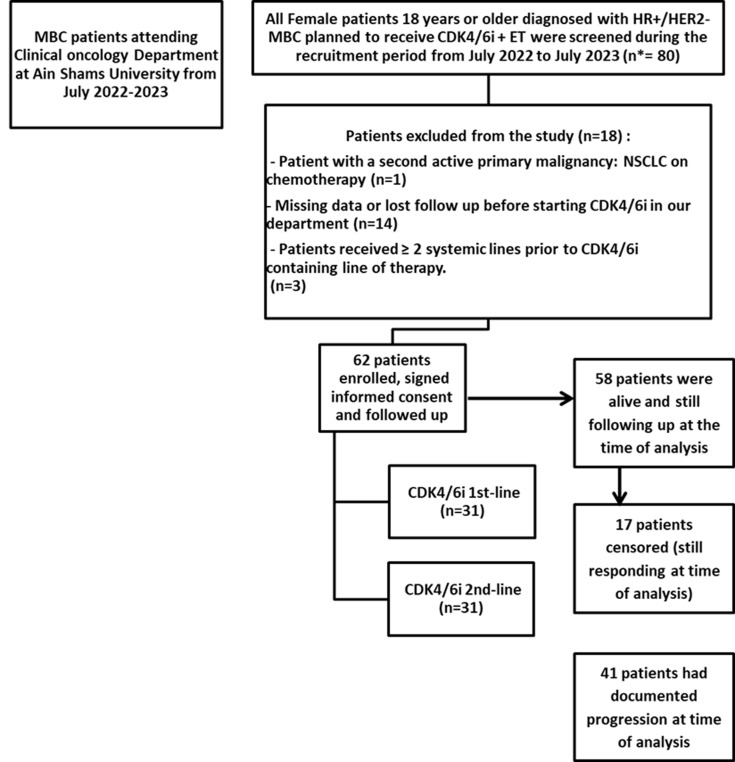
patient population and exclusion criteria

Exclusion criteria included male breast cancer patients and patients with severe organ dysfunction, as determined by clinical signs and symptoms, laboratory abnormalities, or rapidly progressive disease associated with a high risk of life-threatening complications in the short term. Patients who had received prior treatment with any CDK4/6is were excluded. In addition, patients with a history of another malignancy were excluded unless they were in complete remission and had been off anticancer therapy for a minimum of three years (Figure 1).

**Variables:** primary outcomes were to measure efficacy included PFS and OS. Progression-free survival (PFS) was calculated from the time of patient enrollment until documented disease progression, death, or the date of last follow-up, while OS was calculated from the time of enrollment until death. Primary endocrine resistance was defined as recurrence during the first 2 years of adjuvant ET or progression within the first 6 months of first-line ET in the metastatic setting. Secondary endocrine resistance was defined as recurrence during adjuvant ET after the first 2 years or progression after 6 months of first-line ET in the metastatic setting. Secondary outcomes were: objective response rate (ORR), adverse events (AEs) profile, treatment adherence, treatment interruptions, dose reductions, and their impact on efficacy.

**Data sources/measurement:** data were recorded and collected prospectively from patients´ medical records, including baseline demographics, disease characteristics, and treatment details. Estrogen and progesterone receptors (ER and PR) were defined as positive if ≥1% expression was detected. HER2 was defined as low if immunohistochemistry (IHC) was +1 or +2 and negative by silver in situ hybridization (SISH). Laboratory tests, including complete blood count (CBC) and liver and kidney function assessments, were performed before the start of each treatment cycle (every 28 days), while ECGs were performed at baseline and repeated only when clinically indicated. Adverse events were documented at each visit and graded according to NCI CTCAE, version 5.0 [10] prior to each cycle. Hematological AEs were managed with treatment interruptions, and other AEs were treated supportively when needed. Dose reductions (DR), treatment delays, and discontinuation rates were prospectively documented. Adherence was measured based on patients' attendance at clinic visits to receive their treatment. Patient responses were evaluated through radiological imaging, such as computed tomography (CT), positron emission tomography-computed tomography (PET-CT), or magnetic resonance imaging (MRI) for cases of central nervous system (CNS), vertebral, or suspected liver metastases, as determined by the treating physician every 3-4 months. The objective response rate (ORR) was described using RECIST 1.1.

**Bias:** to minimize selection bias, all consecutive eligible patients treated during the recruitment period were included. Standardized institutional protocols were followed for biomarker testing, imaging, and treatment monitoring to reduce information bias.

**Sample size:** using the PASS 15 program for sample size calculation, assuming a prospective observational design with consecutive sampling of eligible patients, setting confidence level at 95% and margin of error at 15%. It was estimated that a sample size of 45 patients was needed to detect an expected PFS rate of 51% [11].

**Participant recruitment:** participants were recruited using a consecutive sampling strategy. Eligible patients were identified by the investigators at the Clinical Oncology Department, Ain Shams University, from the newly referred HR+/HER2- MBC patients planned to receive CDK4/6is+ET. All consecutive eligible women during the study recruitment period were invited to participate and approached in person by the site research staff prior to initiation of CDK4/6is +ET. Written informed consent was obtained from all participants before enrollment according to center protocols, after which patients were prospectively followed according to the study protocol.

**Quantitative variables:** quantitative data, such as age, were presented as means, standard deviations, and ranges for parametric variables, and as medians with Interquartile ranges (IQR) for non-parametric variables such as ECOG PS score.

**Statistical analysis:** data were collected, revised, coded, and entered into the Statistical Package for the Social Sciences (IBM SPSS Statistics, Version 27.0; IBM Corp., Armonk, NY, USA). Variables with missing data were discarded from the analysis. Quantitative data were presented as means, standard deviations, and ranges for parametric variables, and as medians with Interquartile ranges (IQR) for non-parametric variables. Clinical efficacy outcomes included PFS and OS. Progression-free survival (PFS) was defined and calculated from the time of enrollment and initiation of CDK4/6is + ET to documented disease progression or death from any cause, whichever occurred first. OS was defined and calculated as the time from enrollment and treatment initiation to death from any cause. Patients without an event at the time of analysis were censored at the date of last follow-up.

Survival functions for PFS and OS were analyzed using the Kaplan-Meier method. Comparisons between groups were performed using the log-rank test to evaluate the association between PFS and clinicopathological characteristics, treatment line, type of CDK4/6is, and dose modification variables (including dose reductions, treatment interruptions, and treatment delays). Univariate Kaplan-Meier analyses with log-rank testing were used to explore these associations. As no statistically significant associations were identified in univariate analyses, multivariate Cox proportional hazards regression analysis was not performed. Adverse events were assessed descriptively by reporting the incidence and severity of AEs, graded according to NCI CTCAE version 5.0 [10]. Group comparisons for categorical toxicity variables were conducted using the Chi-square test or Fisher´s exact test, as appropriate, when the expected cell count was less than 5. Toxicity and dose modification variables were also descriptively analyzed in relation to treatment exposure. The confidence interval (CI) was set at 95%, and the accepted margin of error was 5%. A p-value of ≥0.05 was considered statistically significant. All analyses were conducted in triplicate to ensure the reliability and reproducibility.

**Ethical consideration:** the study protocol was approved by the Research Ethical Committee, Faculty of Medicine, Ain Shams University, Cairo, Egypt (FWA:000017585/MD148-2022). Written informed consent was obtained prior to the initiation of treatment with CDK4/6is according to institute protocols.

## Results

**Participants**: a total of 80 patients were planned to receive CDK4/6is and initially reviewed for eligibility. Of these, 18 patients fell under the exclusion criteria (Figure 1), leaving 62 patients who were confirmed eligible and were enrolled, followed up, and included in the final analysis.

**Descriptive data:** the mean age was 53.8 years (range: 29 - 85). Thirty-six patients were postmenopausal, and 26 were premenopausal. Forty-six (74.1%) had an ECOG PS of ≤1, and 16 (25.8%) had a PS of ≥2. Fifteen patients were endocrine sensitive (24.2%), 8 had primary endocrine resistance (12.9%), and a significant proportion (62.9%) developed secondary resistance. The cohort included 23 patients with one or more medical comorbidities. Patients´ clinicopathological characteristics are summarized in Table 1.

**Table 1:** baseline demographics and tumor characteristics of study participants, at the time of CDK4/6is initiation, recruited from the Clinical Oncology Department of Ain Shams University Hospitals (Egypt), from July 2022 to July 2023 (N=62)

Baseline clinical characteristics	
**Age**	**Value (n = 62)**
≤40	10 (16.1%)
(41 - 64)	39 (62.9%)
≥65	13 (21.0%)
Mean age ± SD	53.89 ± 12.72
**Menopausal status**	
Premenopausal	26 (41.9%)
Postmenopausal	36 (58.1%)
**ECOG PS**	
0-1	46 (74.1%)
≥2	16 (25.8%)
**Family history**	
irrelevant	55 (88.7%)
Breast cancer	5 (8.1%)
Other malignancies	2 (3.2%)
**ER status**	
ER negative	0 (0%)
ER positive	62 (100%)
**PR status**	
Negative	7 (11.3%)
Positive	55 (88.7%)
**HER2 status**	
0	41 (66.2%)
low	21 (33.8%)
**Medical co-morbidities**	
No	39 (62.9%)
Hypertensive	16 (25.8%)
Diabetic mellitus	12 (19.3%)
Cardiac	3 (4.8%)
Others	10 (16.1%)
**Site of metastatic deposits**	
Bone only	23 (37.9%)
Visceral	39 (62.1%)
CNS	5 (8.1%)
≥2 sites	37 (59.6%)
**Time of diagnosis of MBC**	
De novo metastatic	20 (32.3%)
Progressed on adjuvant	34 (54.8%)
Progressed after adjuvant	8 (12.9%)
**Hormonal resistance**	
Sensitive	15 (24.2%)
One year of resistance	8 (12.9%)
Two-year resistance	39 (62.9%)

SD: standard deviation; ECOG PS: Eastern Cooperative Oncology Group Performance Status; ER: estrogen receptors; PR: progesterone receptors; HER2: human epidermal growth factor receptor 2; CNS: central nervous system; MBC: metastatic breast cancer

Thirty-one patients were treated with CDK4/6is in combination with ET as first-line therapy, and 31 patients received it as second-line following progression on a single ET line. Thirty patients started with Ribociclib; of these, 13 switched to Palbociclib during treatment due to financial reasons (n=12), and Ribociclib-associated cardiac toxicity (n=1). Nine patients started with Abemaciclib; of these, 2 switched to Palbociclib due to financial reasons. Twenty-three patients started with Palbociclib and remained on it till progression or the end of the study. The most common concomitant ET was fulvestrant (n=39). While on CDK4/6is, 28 patients received radiotherapy to the bone for pain palliation. Treatment details are mentioned in Table 2. Patients were followed-up until September 15, 2024, with a median follow-up of 14.2 months.

**Table 2:** subgroup analysis of progression-free survival according to patients’ baseline demographics, disease characteristics, and treatment-related factors (N=62)

Variables		Total N	N of event	PFS (months)		P-value
				Median	S.E	
Age group	≤40	10	7	10	1.491	0.816
	41 - 64	39	25	11	1.187	
	≥ 65	13	9	13	2.996	
ECOG	0 - 1	46	31	12	2.15	0.580
	≥2	16	10	10	3.68	
Medical co-morbidity	No	39	26	13	2.332	0.826
	Yes	23	15	10	1.095	
Menopausal status	Postmenopausal	36	26	14	1.146	0.497
	Premenopausal	26	15	11	3.561	
Hormonal resistance	Sensitive	15	9	14	2.567	0.755
	One year resistance	8	6	6	-	
	Two year resistance	39	26	11	0.869	
CDK4/6i line number	First line	31	19	14	4.128	0.44
	Second line	31	22	11	0.677	
CDK4/6i type	Ribociclib	30	25	10	1.643	0.176
	Palbociclib	23	12	13	-	
	Abemaciclib	9	4	21	5.542	
Concomitant ET with CDK4/6i	Fulvastrant	39	28	10	0.986	0.168
	AI	23	13	14	3.41	
Drug interruptions	No	19	11	11	2.097	0.789
	Yes	43	30	11	1.841	
CDK4/6is dose reduction	No	43	30	12	2.07	0.926
	Yes	19*	11	10	0.83	
Switched to another CDK4/6i	No	47	31	10	0.937	0.102
	Yes	15	10	20	4.508	

* : including the one patient who discontinued ribociclib due to cardiac toxicity; PFS: progression-free survival; S.E.: standard error; ECOG: Eastern Cooperative Oncology Group; CDK4/6i: cyclin-dependent kinase 4/6 inhibitors; ET: endocrine therapy

### Outcome data

#### Clinical efficacy

**Progression-free survival (PFS):** at the time of analysis in September 2024, with a median follow-up time of 14.2 months, 58 patients (93.6%) were alive and still following up, and 41 patients (66.1%) had documented disease progression. The overall median PFS from the start of CDK4/6is therapy to disease progression was 11 months (95%CI: 7.74-14.26), ranging from 6 to 27 months, and the PFS rate at 12 months was 46.3%. For patients receiving CDK4/6is as first-line therapy, median PFS was 14 months (95%CI: 6.36-21.09), while for those receiving it as second-line therapy, PFS was 11 months (95% CI 9.6-14.3).

**Overall survival (OS):** the OS outcomes were not mature at the time of the data cut-off, with only 2 deaths (3.2%). The median OS was not reached in either group.

**Objective response rate (ORR):** the best response observed was a complete radiological response in one patient, while 22 patients (35.5%) had a partial response, and the majority (37.5%) maintained stable disease throughout the follow-up period.

**Dose reductions and treatment delays:** causes and rates of treatment modifications are listed in Table 3. Dose reductions were required in 19 patients (24.6%) due to various toxicities. Toxicity incidence rates and grades are presented in Table 4, which led to interrupted schedules in 43 patients (69.3%). One patient had to discontinue Ribociclib due to decreased Left Ventricular Ejection Fraction (LVEF) and switched to Palbociclib upon recovery after 3 months.

**Table 3:** details of treatment schedule interruptions among the study cohort (N=62), including frequency, duration, and underlying causes such as treatment-related toxicities or patient-related factors

Treatment schedule details		Total no = 62
Dose at starting CDK4/6i	Full dose	62 (100%)
CDK4/6i interruptions	No	19 (30.6%)
	Yes	43 (69.4%)
Causes of interruptions*	Toxicity	35 (81.3%)
	Financial	18 (41.9%)
	Social	3 (7.0%)
	Surgery	1 (2.3%)
	Radiotherapy	1 (2.3%)
Interruption frequencies	0-2 times	30 (69.8%)
	≥3 times	13 (30.2%)
Toxicity led to interruptions**	Neutropenia	26 (74.2%)
	Anemia	4 (11.4%)
	Thrombocytopenia	2 (5.7%)
	Elevated liver enzymes	2 (5.7%)
	Hypocalcaemia	1 (2.8%)
	Decreased left ventricular ejection fraction (LVEF)	1 (2.8%)
	Skin toxicity	1 (2.8%)
	Diarrhea	1 (2.8%)
	Arrhythmia	2 (5.7%)
	Fatigue	1 (2.8%)
	Elevated s. creatinine	1 (2.8%)
Longest delay due to toxicity per episode in days	Median (IQR)	7 (7 - 10)
	Range	3 - 90
Longest delay due to any cause per episode in days	Median (IQR)	30 (7 - 60)
	Range	3 - 90

* : 15 patients had ≥2 causes leading to treatment delay; **: 6 patients suffered from ≥2 adverse effects that led to treatment delay duration, and underlying causes such as treatment-related toxicities or patient-related factors; CDK4/6i: cyclin-dependent kinase 4/6 inhibitors

**Table 4:** adverse events profile of CDK4/6 inhibitors among the study cohort (N=77), detailing the type, severity of treatment-related toxicities, and the need for dose reduction

Adverse event type		N = 77*		Grade 1-2		Grade 3-4	
**Hematological toxicity**							
Neutropenia		50	64.9%	25	50 %	25	50 %
Thrombocytopenia		6	7.8%	5	83.3%	1	16.7%
Anemia		21	27.3%	18	85.7%	3	14.3%
DR**		14	(18.1%)				
**Non-hematological toxicities**							
QT prolongation		1	1.3%				
DR**	Yes	1	100%				
Left ventricular ejection fraction drop		1	1.3%				
DR**	Omitted	1	100%				
Fatigue		19	24.7%	18	94.7%	1	5.3%
Nausea		7	9.1%	7	100.0%		
Infection		5	6.5%	5	100%		
Headache		9	11.7%	9	100%		
Diarrhea		15	19.5%	14	93.3%	1	6.7%
Stomatitis		9	11.7%	9	100%		
Dyspnea		5	6.5%	5	100%		
Pyrexia		4	5.2%	4	100.0%		
Myalgia		5	6.3%	5	100.0%		
Muscle spasms		3	3.8%	3	100.0%		
Abdominal pain		5	6.3%	5	100.0%		
Rash		4	5.2%	4	100.0%		
Arthralgia		7	9.1%	7	100.0%		
Anorexia		6	7.8%	6	100%		
Creatinine elevation		8	10.4%	8	100%		
DR**	Yes	1	12.5%				
Elevated liver enzymes		15	19.0%	13	86.7%	2	13.3%
DR**	Yes	1	6.7%				
Constipation		5	6.5%	5	100.0%		
Alopecia		8	10.4%	8	100%		
Vomiting		5	6.8%	5	100%		
Pneumonitis		1	1.3%	1	100%		
Hot flush		8	10.4%	8	100%		
RBS elevation		2	2.5%	2	100%		
electrolytes imbalance		9	11.7%	9	100%		
Skin pigmentation		2	2.6%	2	100.0%		
Hyperlipidemia		3	3.8%	3	100.0%		
Skin dryness		5	6.3%	4	80%	1	20%
**DR	Yes	1	20.0%				
Neuropathy		3	3.8%	3	100.0%		

* : 15 patients switched to Palbociclib, they are added to the total population, making the total number 77; **DR: dose reduction; CDK4/6i: cyclin-dependent kinase 4/6 inhibitors

**Adverse events (toxicity):** the main toxicities observed were hematological, with neutropenia of any grade occurring in 64.9% of patients; grade 3-4 AEs were reported in 32.4%. Fatigue was the most common documented non-hematological AE, reported in 24.7% of patients. Data on AEs are presented in Table 4. In the Ribociclib and Palbociclib groups, the most common AE of any grade was neutropenia (70.0% and 68.4%, respectively), followed by fatigue in 8 patients (26.7%) in the Ribociclib group and 10 patients (26.3%) in the Palbociclib group. All reported AEs resolved with either treatment interruption or supportive care.

**Impact of clinicopathological factors, type of CDK4/6is, and dose modifications on treatment outcomes:** the results indicated that patient characteristics, including age, menopausal status, PR status, HER2 status, ECOG PS score, medical comorbidities, and type of endocrine resistance, did not significantly influence PFS. Subgroups’ analysis is shown in Table 2. Furthermore, the type of CDK4/6is administered, concomitant ET, and treatment regimen alterations, such as delays, dose reductions, or switching to another CDK4/6is, did not significantly affect treatment efficacy.

**Impact of clinicopathological factors, type of CDK4/6is and radiotherapy on adverse events profile:** the incidence of neutropenia and elevated serum creatinine levels was directly correlated with age, being more prevalent in patients over 40 years old (p=0.032 and p=0.007, respectively). Additionally, patients with medical comorbidities had significantly greater severity of hematological AEs, including grade 3-4 anemia and thrombocytopenia (p=0.02 and p=0.05, respectively). The occurrence and severity of other AEs were not influenced by patients´ clinicopathological characteristics. Concurrent palliative radiotherapy, administered to 28 patients (45.1%), did not significantly impact the incidence or severity of hematological AEs associated with CDK4/6is use.

### Other analyses

**Subsequent treatment after progression on CDK 4/6i:** upon progression on CDK4/6is, most of the patients (12) were shifted to Everolimus and Concomitant ET line (29.2%), nine patients (14.5%) received chemotherapy (Paclitaxel single agent=4, Paclitaxel/Carboplatin=1, Capecitabine=2, Gemcitabine=1, Docetaxel=1), one patient was shifted to Fulvastrant, and 19 patients lost follow-up.

## Discussion

This real-world study included 62 patients and demonstrates that CDK4/6 inhibitors are both safe and effective in real-life clinical settings. The results are consistent with other RWD studies, though some discrepancies were observed compared to RCTs. In the first-line treatment group, the median PFS was lower than in the pivotal trials [12-14]. However, the second-line treatment group showed a median PFS similar to that observed in RCTs investigating CDK4/6is in subsequent lines, such as the 11.2-month median PFS reported in PALOMA-3 for Palbociclib [15] and the 8-month median PFS reported in the SONIA trial for second-line CDK4/6is use. The SONIA trial, which included a large sample size, demonstrated that there was no significant difference in PFS2 between first- and second-line CDK4/6is administration [16], providing solid evidence that CDK4/6is treatment can be safely delayed to the second-line in settings with limited access, such as ours.

The differences in PFS outcomes can be attributed to several factors. Our cohort consisted of a heterogeneous, unselected population with a median age of 53.8 years, including 25.8% of patients with an ECOG PS ≥2. Additionally, a significant portion of our population (75.8%) had endocrine-resistant disease, either primary (12.9%) or secondary (62.9%), having progressed on prior ET. These patients, along with those with established medical comorbidities, are typically excluded from RCTs [12-14]. Moreover, the presence of visceral metastatic disease, including CNS metastases, was common in our cohort and is known to be associated with a poorer prognosis [17,18], further contributing to the shorter PFS observed in this real-world setting.

Regarding RWD, obtaining comprehensive clinical characteristics for each study reviewed was challenging due to limited data availability, with some studies only providing abstracts. The PFS results from our study were comparable to those conducted in the USA [19], China [20], Sweden [21], Brazil [22], and Saudi Arabia (KSA) [23]. Notably, our results exceeded those reported in India (PFS 7.7 months) [24] and Slovenia (PFS 8.3 months) [25]. In these RWD, Palbociclib was the predominant CDK4/6is used, and the majority reported a younger median patient age, similar to our study. In contrast to our cohort, RWD from UK [26], Australia [27], Germany [28], and Jordan [29], where CDK4/6is were mainly used in de novo MBC or where Ribociclib predominated, reported longer PFS aligning with MONALEESA-2 results [13]. The differences in PFS outcomes could be attributed to variations in the treatment setting and the type of CDK4/6is used. A comparative study evaluating the three major CDK4/6 inhibitors could offer clearer insights into their relative efficacy.

The toxicity profile observed in our cohort was consistent with those reported in RCTs and other RWD. As anticipated, the most common toxicities were neutropenia in patients receiving Palbociclib or Ribociclib and diarrhea in those receiving Abemaciclib. Interestingly, the rate of DRs in our study was lower than that reported in both RCTs and other RWD, with only 24.6% of patients requiring a DR. In comparison, the reported DR rates were 36% in PALOMA-2 [14], 45% in the MONALEESA trials [30], and 46.5% in MONARCH3 [12], and in RWD studies, where nearly half of the patients required DRs [25-27,31,22]. The lower rate of DRs in our cohort may be attributed to the generally mild nature of the AEs experienced, which were effectively managed through treatment interruptions and supportive care. Additionally, concurrent bone radiotherapy did not increase hematological toxicity, aligning with Sönmez *et al*. (2021), who found CDK4/6is well-tolerated with palliative or ablative radiotherapy [32].

Dose reduction, patients´ characteristics, and treatment interruptions did not significantly impact PFS (HR=1; 95% CI: 0.5-1.9) in our cohort; a finding may be partially attributed to the small sample size but is supported by the findings of Kristensen *et al*. who also reported that CDK4/6is dose reduction did not impact PFS [33]. This suggests that more flexible treatment approaches, such as dose titration or longer treatment breaks, could be explored in future studies, particularly for vulnerable populations who were found to be at higher risk for treatment-related toxicities as revealed by our data and other RCTs [34,35]. Such strategies may be particularly beneficial in low-income countries with limited access to continuous therapy.

Our study had several limitations; despite including all eligible patients who received CDK4/6is at our center since July 2022, the sample size remained limited. Additionally, the median follow-up was relatively short, limiting the number of observed events. Larger, multicenter studies are needed to validate and confirm our findings in this population.

## Conclusion

To the best of our knowledge, and despite certain limitations, our findings offer valuable RWD insights into the use of CDK4/6 inhibitors in HR+/HER2- MBC patients in Egypt. The study highlights the need for further investigations into population-specific factors that may influence treatment outcomes, such as genetic variability and comorbidities. The results demonstrate that CDK4/6 inhibitors are effective, well-tolerated, and convenient for routine clinical use, with a manageable toxicity profile. Importantly, our data suggest that DRs and treatment interruptions do not adversely affect PFS, supporting further prospective studies into the efficacy of lower-dose regimens.

### What is known about this topic

- CDK4/6is first-line improve PFS in MBC;
- CDK4/6is can be given in secondary hormonal resistance.

### What this study adds

- Real-world data from Africa regarding response to CDK4/6is;
- Treatment irregularity and dose reduction in LMIC are possible with similar PFS.
